# Purification, characterization, and enzyme kinetics of a glutathione S transferase from larvae of the camel tick *Hyalomma dromedarii*

**DOI:** 10.1186/s43141-023-00486-w

**Published:** 2023-03-08

**Authors:** Hassan M. M. Masoud, Mohamed S. Helmy, Doaa A. Darwish, Mahmoud A. Ibrahim

**Affiliations:** 1 Molecular Biology Department, National Research Centre, El-Tahrir St, Dokki, Giza, Egypt; 2 Proteome Research Laboratory, Central Laboratories Network and Centers of Excellence, National Research Centre, El-Tahrir St, Dokki, Giza, Egypt

**Keywords:** Glutathione-S-transferase, Purification, Characterization, Camel tick, *Hyalomma dromedarii*

## Abstract

**Background:**

Glutathione s-transferases (GSTs) perform an essential role in detoxification of xenobiotics and endogenous compounds via their conjugation to reduce glutathione.

**Results:**

A GST enzyme, designated tick larvae glutathione S transferase (TLGST), was purified from larvae of the camel tick *Hyalomma dromedarii* via ammonium sulfate precipitation, glutathione-Sepharose affinity column and Sephacryl S-300 chromatography. TLGST-specific activity was found to be 1.56 Umg^−1^ which represents 39 folds and 32.2% recovery. The molecular weight of TLGST purified from camel tick larvae was found as 42 kDa by gel filtration. TLGST has a *pI* value of 6.9 and was found a heterodimeric protein of 28 and 14 kDa subunits as detected on SDS-PAGE. The Lineweaver–Burk plot calculated the *km* for CDNB to be 0.43 mM with *Vmax* value of 9.2 Umg^−1^. TLGST exhibited its optimal activity at pH 7.9. Co^2+^, Ni^2+^ and Mn^2+^ increased the activity of TLGST while Ca^2+^, Cu^2+^, Fe^2+^ and Zn^2+^ inhibited it. TLGST was inhibited by cumene hydroperoxide, *p*-hydroxymercuribenzoate, lithocholic acid, hematin, triphenyltin chloride, *p*-chloromercuribenzoic acid (*p*CMB), N-p-Tosyl-L-phenylalanine chloromethyl ketone (TPCK), iodoacetamide, EDTA and quercetin. *p*CMB inhibited TLGST competitively with *Ki* value of 0.3 mM.

**Conclusions:**

These findings will help to understand the various physiologic conditions of ticks and targeting TLGST could be significant tool for development of prospective vaccines against ticks as a bio-control strategy to overcome the rapid grows in pesticide-resistant tick populations.

**Graphical Abstract:**

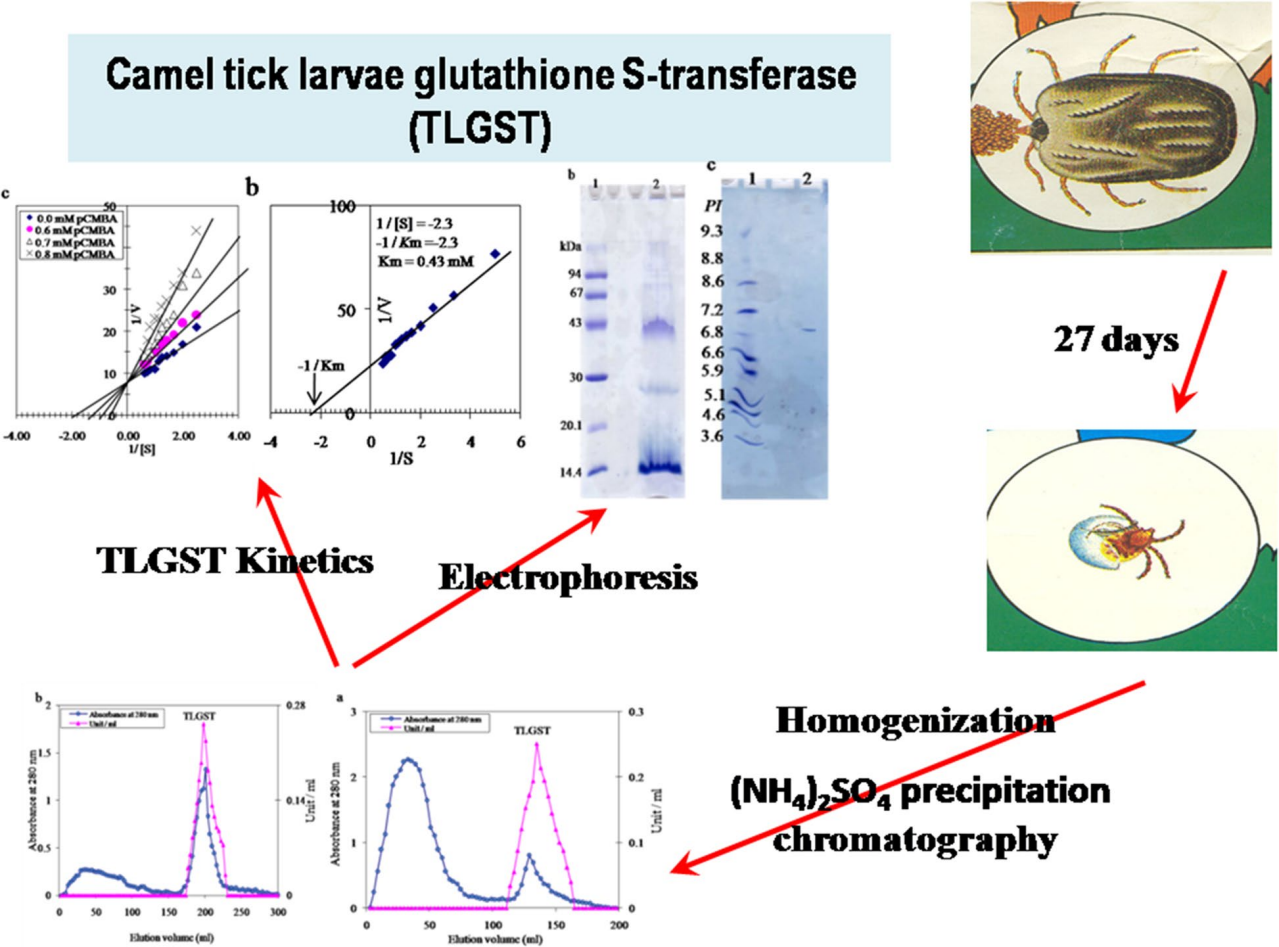

## Background

Camels produce meat, milk, wool, hair and hide and are utilized in transport and most of which are infected with the external parasites ticks [4, 39]. Ticks are blood-sucking parasitic arachnids infesting humans and all animals of economic importance leading to big losses in livestock production through transmission of various pathogenic organisms comprising rickettsiae, bacteria, protozoa, spirochetes and viruses. Ticks cling to animal hides and then introduce the toxins into them and transmit the diseases, which reduce their productivity [14, 32]. The ticks *Hyalomma dromedarii* are specific on camels and infecting them with the spring–summer encephalitis virus [3]. Studies about tick behaviour, ecology and physiology provide well understanding about these species, making them important tools for developing new biocontrol methods. Studying of tick physiology has acquired growing significance regarding the mechanisms implicated in toxins detoxification [15, 20, 27]. Ticks are exposed to various oxidative stress causing agents like exposure to acaricides and the ability for ingesting large quantities of blood from their hosts. Various potential toxic molecules are included in blood which have the ability to elevate the output of various free radicals such as reactive oxygen species (ROS) causing oxidative stress [16, 48]. Ticks must counteract the ROS produced by uptaking and digesting blood meals. Accordingly, ticks utilize various antioxidant molecules like superoxide dismutase (SOD) [25], catalase (CAT) [23, 28], glutathione peroxidase (GPx) [24] and glutathione S-transferases (GSTs) to overcome oxidative stress [21].

Glutathione S-transferase (GST) [GSTs, EC 2.5.1.18] is an enzyme that acts to excrete physiologic and xenobiotic molecules for cells protection from chemical toxins and stresses [9, 15]. GSTs are ubiquitous enzymes of multiple functions exist in microbial, plant and animal sources. The primary function of GSTs is detoxifying different toxins (insecticides, herbicides, drugs) via catalyzing the conjugation to glutathione (GSH). GSH is a fundamental tri-peptide sulfhydryl antioxidant found in plant and animal tissues in very low concentrations for performing the function of detoxification of xenobiotics and endogenous oxidants [17, 33, 34]. This takes place by the nucleophilic attack of the GSH sulfur atom on electrophilic substrates,decreasing their reactivity with cellular macromolecules and forming an important line of defence for cell components from reactive molecules [22]. GSTs are divided into soluble cytosolic (alpha, mu, Pi, omega, theta, delta, sigma and zeta), mitochondrial GST (alpha, mu, Pi and kappa), and membrane bound microsomal GST [7, 49]. A special property of all cytosolic GSTs is being homo- or heterodimers with two substrate binding sites: a highly specific and conserved G-site (GSH binding site) and H-site (hydrophobic binding site) for electrophilic substrates [30]. The inhibition mechanisms of GST by different inhibitor compounds like quercetin, hematin, ethacrynic acid and triphenyltin chloride have been broadly studied as a diagnostic way for resistance mechanisms [2, 11, 40, 46]. GSTs of various tick species were identified and characterized; *Rhipicephalus microplus* [38], *R. appendiculatus* [8], *Dermacentor variabilis* [10], *R. Sanguineus* [12] and *H. longicornis* [21]. The aim of this study is purification and characterization of GST enzyme from *H. Dromedarii* larvae and inspection of the effect of some chemical inhibitors on the tick GST activity which will be helpful for development of prospective vaccines against ticks.

## Methods

### Tick material

The engorging females of camel tick *H. dromedarii* were gathered from a market for camels near Cairo and incubated at 28 ºC and 85% relative humidity. Eggs were daily collected from oviposited females, immediately frozen (− 40 °C) or either held at same conditions till the required age then frozen and stored at 3 days interval (0, 3, 6, 9 etc.). The larvae hatched after 27 days.

### Chemicals

1-chloro-2, 4-dinitrobenzene (CDNB), phenylmethylsulfonylfluoride (PMSF), reduced glutathione (GSH), glutathione sepharose 4 fast flow, sephacryl S-300, cumene hydroperoxide, *p*-hydroxymercuribenzoate, lithocholic acid, hematin, *p*-chloromercuribenzoic acid (*p*CMB), N-p-tosyl-l-phenylalanine chloromethyl ketone (TPCK), iodoacetamide, gel filtration molecular weight markers, and triphenyltin chloride were purchased from Sigma Chemical Co. All other chemicals were of analytical grade.

### Assay of GST activity

The GST assay reaction mixture contained in 1 ml total volume of 0.1 M K-phosphate buffer, pH 6.5, 1 mM 1-chloro-2,4-dinitrobenzene (CDNB), 1 mM GSH, and enzyme solution. The increase in absorbance was monitored at 340 nm for 3 min at 25 °C. The product extinction coefficient was considered as 9.6 mM^−1^ cm^−1^. One GST unit is equivalent to the enzyme amount which conjugates 1 μmole CDNB in 1 min [19].

### Protein determination

Protein concentrations were estimated via the dye binding procedure [6] using albumin from bovine serum (BSA) as a standard.

### Purification of camel tick larvae glutathione S-transferase

#### Preparation of crude extract

All experimental procedures were carried out at 4 °C. Homogenization of camel tick larvae was taken place in 0.02 M Na-phosphate buffer pH 7.0, using a Teflon pestled homogenizer. Cell residues and indissoluble substances were brought out via centrifugation (12,000 × *g*, 30 min) and supernatant was kept as crude extract.

#### Ammonium sulfate treatment

Crude extract was gradually saturated with 40% (NH_4_)_2_SO_4_ with continuous stirring (30 min, 4 °C) followed by centrifugation (8000 × *g*, 20 min). The precipitate was ignored and the supernatant was raised to 80% (NH_4_)_2_SO_4_ saturation, obtaining the pellet via centrifugation (12,000 × *g*, 30 min) and dissolving in 0.02 M Na-phosphate buffer pH 7.0 followed by inclusive dialysis in this buffer.

#### Glutathione sepharose affinity chromatography

The dialyzed 40 − 80% ammonium sulfate sample was applied onto a GSH-sepharose 4 fast flow column (8 × 1.4 cm) pre-equilibrated with 0.02 M Na-phosphate buffer pH 7.0 and set at a flow rate of 30 ml/h. Washing the column with 100 m1 equilibration buffer and elution of the adsorbed proteins was achieved with 10 mM GSH contained in 0.05 M Tris–HCl buffer pH 8.0. Collection of 3 ml fractions and those with GST activity were combined and concentrated via lyophilization.

#### Sephacryl S-300 size exclusion chromatography

Fractioning the GST containing concentrate on a Sephacryl S-300 column (142 cm × 1.75 cm) pre-equilibrated and run with 0.02 M Na-phosphate buffer pH 7.0 at 30 ml/h flow rate with collection of 2 ml fractions. For estimation of the native GST molecular weight, Sephacryl S-300 column was calibrated with 440 kDa ferritin, 240 kDa catalase, 150 kDa alcohol dehydrogenase, 67 kDa bovine serum albumin, and 17 kDa myoglobin as standard markers.

### Electrophoretic analysis

Twelve percent SDS PAGE was done for estimation of the purified GST subunits [29, 42]. Isoelectrofocusing PAGE was done for estimation of the *pI* values [35, 41]. Staining of proteins was achieved with 0.25% Coomassie Brilliant Blue R-250.

#### Optimum pH determination

For estimation of the action of pH on GST activity, the enzyme was assayed utilizing two buffers; 0.1 M potassium phosphate (5.7 − 8.0) and Tris–HCl (8.0 − 9.2) at 25 °C.

#### Enzyme kinetics

Michalis-Menten constant (*Km*) was deduced from the Linewear-Burk plot construction utilizing (0.1 − 2 mM) CDNB as substrate at 25 °C.

#### Effect of divalent cations and inhibitors on GST activity

Effect of various cations and inhibitors on GST activity was achieved via preincubation of the purified enzyme with each molecule at 25 °C prior to assaying activity.

## Results

### Purification of tick larvae GST enzyme

**Fig. 1:**
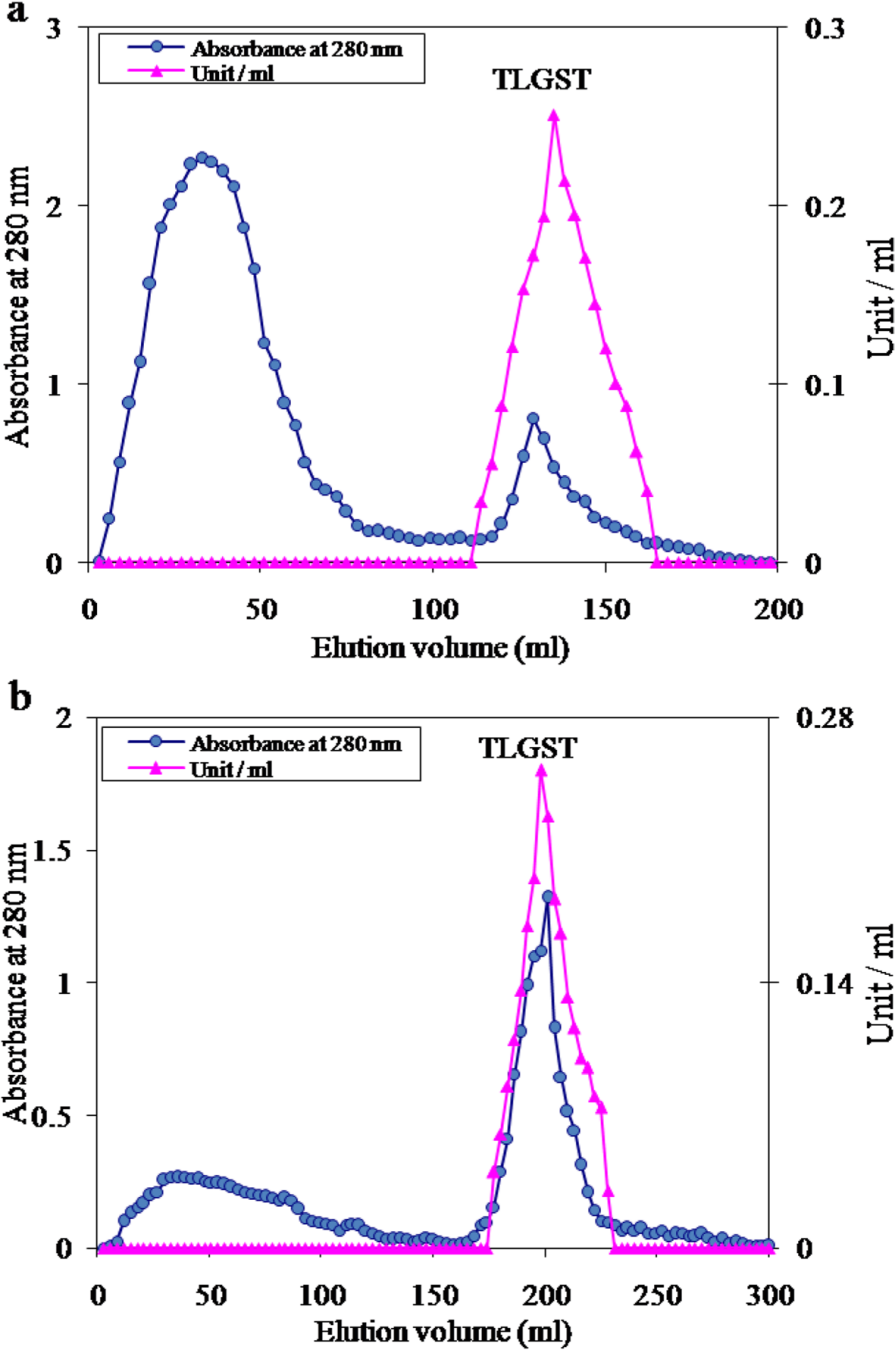
**a** The chromatographic pattern of 40–80% ammonium sulfate fraction of tick larvae crude extract on GSH-Sepharose column (8 × 1.4 cm) previously equilibrated and washed with 0.02 M Na-phosphate buffer pH 7.0. **b** The chromatography of tick larvae TLGST on Sephacryl S-300 column (142 cm × 2.4 cm) formerly equilibrated with 0.02 M Na-phosphate buffer pH 7.0

Typical GST enzyme purification scheme from larvae of *H. dromedarii* tick (Table 1) showed the primary GST-specific activity in larvae crude extract as 0.04 Umg^−1^. Forty to 80% ammonium sulfate precipitated most of GST enzyme and achieved 82.9% recovery. The glutathione sepharose affinity chromatography of 40–80% ammonium sulfate fraction (Fig. 1a) appeared one GST activity peak eluted with 10 mM GSH and designated TLGST. The TLGST concentrate fractionation on Sephacryl S-300 column (Fig. 1b) gives one GST peak with an increase in TLGST-specific activity to 1.56 Umg^−1^, 39 folds and 32.5% yield (Table 1). Apparent molecular weight of *H. dromedarii* TLGST was derived from its elution volume from the Sephacryl column as 42 kDa (Fig. 1b).

**Table 1:** A typical purification scheme of the camel tick larvae GST (TLGST)

Purification step	Total protein (mg)	Activity (unit)	Specific activity	Recovery (%)	Fold purification
Camel tick larvae crude extract	322	12.9	0.04	100	1.0
40–80% (NH_4_)_2_SO_4_ fraction	118	10.7	0.09	82.9	2.3
Glutathione Sepharose fraction	11.6	6.6	0.57	51.0	14.3
Sephacryl S-300 fraction	2.7	4.2	1.56	32.5	39.0

### Electrophoretic analysis of TLGST

**Fig. 2:**
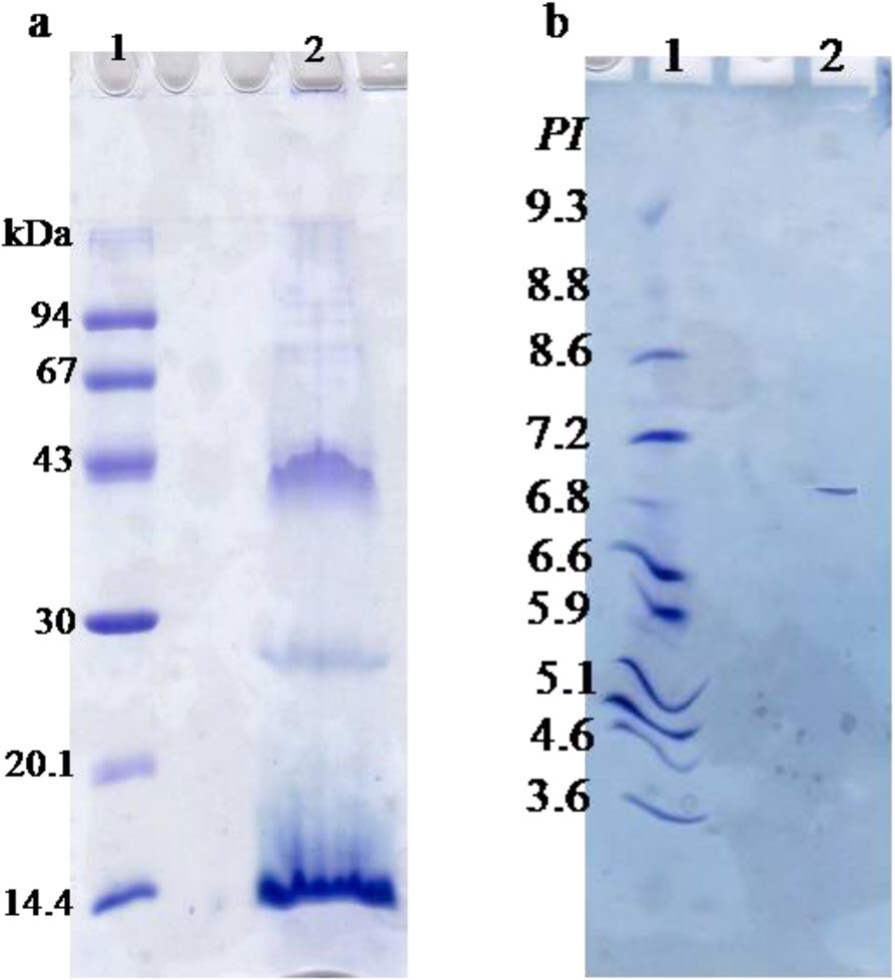
**a** Analysis of TLGST on 12% SDS PAGE; (1) molecular weight marker proteins, (2) TLGST. **b** Analysis of TLGST on isoelectric focusing PAGE: (1) *pI* marker proteins, (2) TLGST

SDS-PAGE of denatured *H. dromedarii* TLGST enzyme (Fig. 2a) displayed residues of the native intact protein (42 kDa) and two protein bands of 28 kDa and 14 kDa representing the TLGST subunits. The TLGST *pI* value was seen as single species on isoelectrofocusing PAGE at pH 6.9 (Fig. 2b).

### Determination of optimum pH and Km value of TLGST

**Fig. 3:**
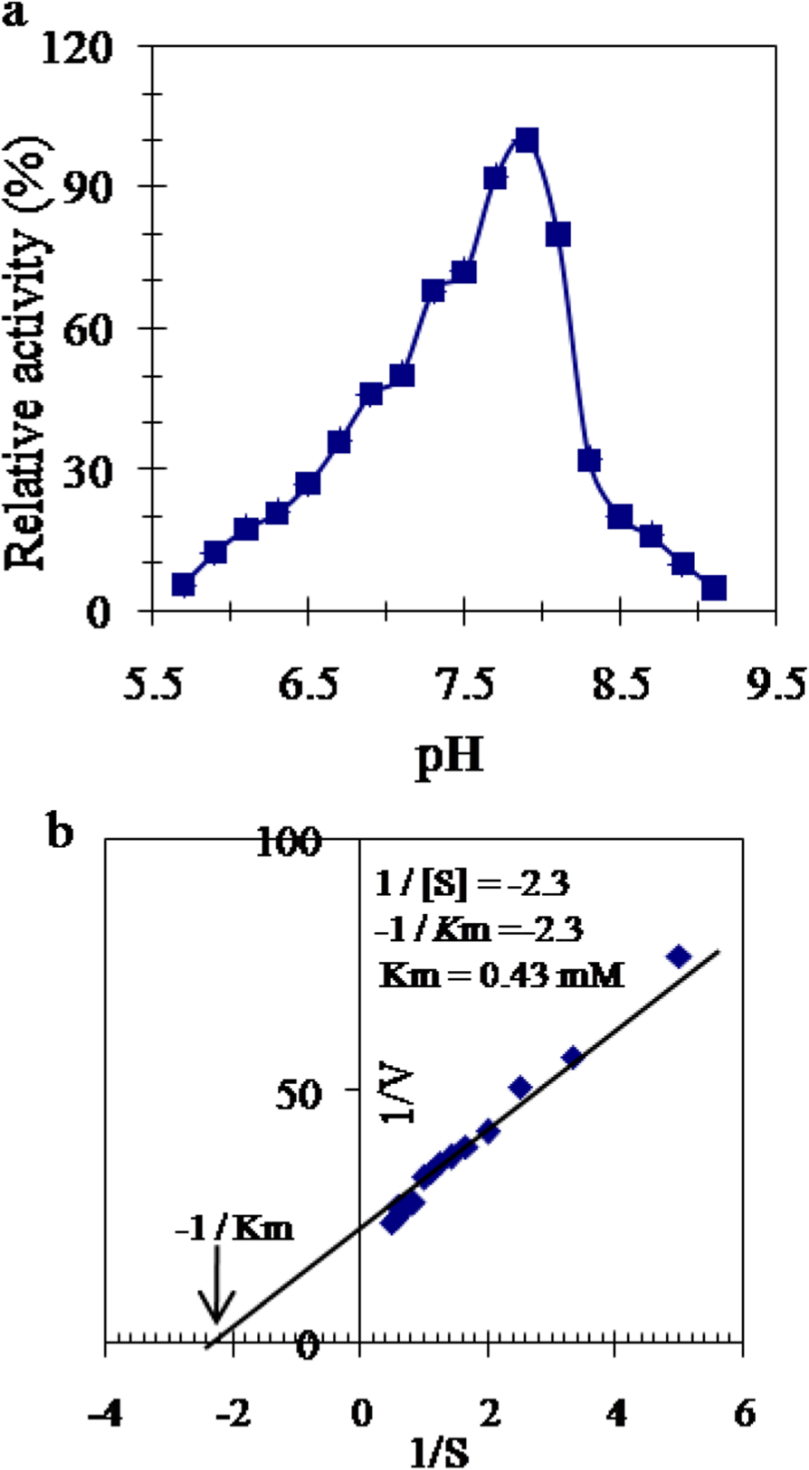
**a** The pattern of TLGST pH profile utilizing 0.1 M potassium phosphate (5.7–8.0) and Tris–HCl (8.0–9.2). **b** Lineweaver–Burk plot of TLGST reaction speed in response to CDNB concentrations

The impact of pH on TLGST activity was tested using K-phosphate and Tris–HCl buffers which revealed the highest activity of the enzyme at pH 7.9 (Fig. 3a). Construction of Lineweaver–Burk plot of reaction speed and substrate concentration reciprocals showed the *Km* value of TLGST as 0.43 mM CDNB with a corresponding *Vmax* of 9.2 Umg^−1^ (Fig. 3b).

### Effect of divalent cations and inhibitors on TLGST

The data present in Table 2 showed TLGST activity with 2 mM and 5 mM of various cations. CoCl_2_, NiCl_2_ and MnCl_2_ led to increasing of TLGST activity while CaCl_2_, CuCl_2_, ZnCl_2_ and FeCl_2_ caused an activity decrease. The influence of various inhibitors on the purified camel tick larvae TLGST is presented in Table 3. N-p-Tosyl-L-phenylalanine, Quercetin and *p*-chloromercuribenzoic acid (*p*CMB) were found the most potent inhibitors of TLGST.

**Table 2:** Effect of divalent cations on the purified TLGST

Reagent	Concentration (mM)	Relative activity (%)
Control	––-	100.0
CaCl_2_	2.0	49.6
	5.0	19.6
CoCl_2_	2.0	114.4
	5.0	134.0
CuCl_2_	2.0	11.4
	5.0	2.6
FeCl_2_	2.0	66.0
	5.0	52.0
MgCl_2_	2.0	100.0
	5.0	76.8
NiCl_2_	2.0	106.6
	5.0	158
ZnCl_2_	2.0	80.2
	5.0	48.0
MnCl_2_	2.0	121.0
	5.0	136.0

**Table 3:** Effect of inhibitors on the purified TLGST

Inhibitor	Concentration (Mm)	Inhibition %
Control	–	0.0
*p*-Chloromercuribenzoic acid (*p*CMB)	1 mM	97.2
*p*-Hydroxymercuribenzoate (*p*HMB)	5 mM	21.2
N-*p*-Tosyl-L-phenylalanine chloromethyl ketone (TPCK)	5 mM	74.8
Dithiothreitol (DTT)	5 mM	10.4
EDTA	5 mM	37.9
β-Mercaptoethanol	5 mM	10.7
Iodoacetamide	5 mM	63
Potassium cyanide	5 mM	2.1
Quercetin	5 mM	88.6
Cumene hydroperoxide	5 mM	16.5
Hematin	5 mM	42.7
Lithocholic acid	5 mM	30.0
Triphenyltin chloride	5 mM	42.7

## Inhibition kinetic of TLGST by *p*CMB

**Fig. 4:**
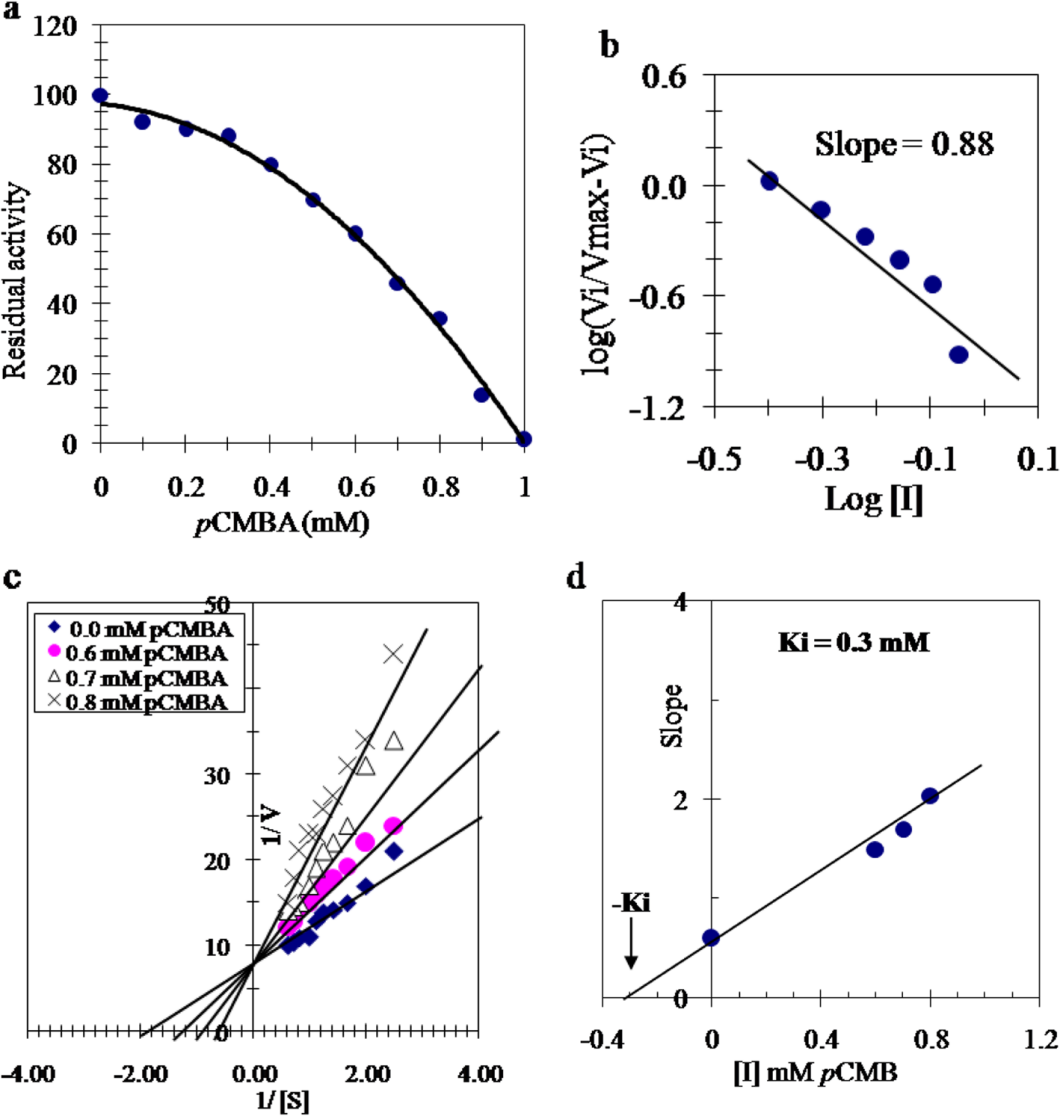
**a** TLGST Inhibition with *p*CMB various concentrations. **b** Hill plot of TLGST inhibition with *p*CMB. **c** Lineweaver–Burk plots showing TLGST inhibition type with *p*CMB. **d** TLGST inhibition constant (*Ki*) value for *p*CMB

The effect of *p*-Chloromercuribenzoic acid (*p*CMB) concentrations on the purified camel tick larvae TLGST indicted that I50 = 0.7 mM *p*CMB with a maximum inhibition of 97.2% was carried out by 1 mM *p*CMB (Fig. 4a). A linear relation was monitored via construction of the Hill plot for TLGST inhibition with *p*CMB giving a slope of 0.88 (Fig. 4b). The type of inhibition of TLGST by *p*CMB was constructed (Fig. 4c) and *Ki* value of TLGST inhibition is determined as 0.3 mM (Fig. 4d).

## Discussion

Glutathione S-transferase is the major detoxifying enzyme that implicated in toxins and insecticide resistance in various insects [13]. Our purpose here was to purify and characterize GST enzyme from larvae of the camel tick *H. dromedarii*. We purified the GST from *H. dromedarii* tick larvae via ammonium sulfate precipitation, GSH-sepharose affinity chromatography and size exclusion chromatography. Similar purification procedures of GSTs were reported, GST from German cockroach [46], from non-biting midges Chironomidae larvae [47] from filarial worms [2], from Liposcelis insects [43] and from blueberry fruits [5]. In this study, the affinity chromatographic pattern of tick larvae GST on GSH-Sepharose column showed one GST activity peak eluted with 10 mM GSH and named TLGST (Fig. 1a). A successive chromatographic step of TLGST via Sephacryl S-300 column (Fig. 1b) gave one GST activity peak and raised TLGST-specific activity to 1.56 Umg^−1^ which correspond 39 folds and 32.5% yield (Table 1). A broad purification folds and recovery percentages for GST from various sources were stated from German cockroach (52.8 folds, 33% yield) [46], from *Atactodea striata* snail (43.2 folds) [45], from *Apis mellifera macedonica* bee (51.9 folds, 54.5% yield) [37], from filarial worms (43.2 folds, 11.1% yield) [2], from Liposcelis insects (32.2, 99.8 and 42.5 folds and 41.7%, 120.8% and 79.2% yields) [43] and from blueberry fruits (189 fold, 23.3% yield) [5].

The elution volume of TLGST from the size exclusion column calculated its native molecular mass as 42 kDa (Fig. 1b). This was assured via SDS-PAGE of denatured purified TLGST (Fig. 2a) which showed ruminants of the native intact protein (42 kDa) and two subunits of 28 kDa and 14 kDa protein bands denoting that TLGST is heterodimeric protein. Various structures were stated for GSTs; monomeric GSTs from the spider mite with Mwt of 22.1 kDa [26], the *Aedes aegypti* mosquito with Mwt of 25 kDa [31] and rice moth with Mwt of 23 kDa [18]. Homodimeric GSTs as that of German cockroach with 25.5 kDa subunit [46], *Atactodea striata* snail with 24 kDa subunit [45], grasshopper insect with 19 kDa subunit [1], filarial worms with 24.6 kDa subunit [2] and teleost *Monopterus albus* fish with 26 kDa subunit [22]. Heterodimeric GSTs as that of *Apis mellifera macedonica* bee with 29 and 25 kDa subunits [37] and *Rhipicephalus microplus* tick with 30.7 and 28.4 kDa [38]. In this present study, TLGST purity was inspected through analysis on isoelectrofocusing PAGE which appeared as one species (Fig. 2b) with *pI* value of 6.9 denoting an alkaline isoenzyme. Various isoelectric points (*pI)* values were reported for GSTs; non-biting midges Chironomidae larvae and *Atactodea striata* snail GSTs *pI* value of 5.5 [45, 47], *Rhipicephalus microplus* tick GST *pI* value was 8.6 [38], *R. Appendiculatus* tick GST *pI* values were 7.67 and 8.51 [8], *Apis mellifera macedonica* bee GSTs *pI* values were 7.4 and 4.58 [37], silkmoth insect GST *pI* value was 6.01 [44] and *H. longicornis* tick GST *pI* value was 7.67 [21].

The camel tick larvae TLGST displayed its optimum pH at 7.9 (Fig. 3a) confirming its alkaline property. The optimum pH of the *H. longicornis* tick GST was around 7.5–8.0 [21], at pH 8.0 in non-biting midges Chironomidae larvae and *Atactodea striata* snail GSTs [45, 47], around 6.5–7.5 in filarial worms GST [2] and 7.2 for blueberry fruits GST [5]. TLGST showed *Km* value of 0.43 mM CDNB and has *Vmax* of 9.2 units/mg protein (Fig. 3b) indicating its high ability to conjugate with CDNB. The GSTs *Km* values for CDNB were 0.82 and 0.64 mM in *H. longicornis* tick HlGST and HlGST2 [21], 0.16 mM in German cockroach [46], 0.62 mM in *Apis mellifera macedonica* bee larvae [37], 2.5 mM in filarial worms [2] and 5.68 mM in blueberry fruits CDNB [5]. In the current study, Co^2+^, Ni^2+^ and Mn^2+^ cause increases in TLGST activity while Ca^2+^, Cu^2+^, Fe^2+^ and Zn^2+^ decreased it (Table 2). The inhibition of TLGST by Cu^2+^ and Zn^2+^ ions was consistent with that of Van fish GST [36]. The effect of various inhibitors on the purified TLGST (Table 3) showed slight inhibition of TLGST with DTT, β-Mercaptoethanol, Cumene hydroperoxide and *p*-Hydroxymercuribenzoate (*p*HMB). It was moderately inhibited with Lithocholic acid, Hematin and Triphenyltin chloride. TLGST was potently inhibited with the cysteinyl protease inhibitors *p*-chloromercuribenzoic acid (*p*CMB) and N-p-Tosyl-L-phenylalanine chloromethyl ketone (TPCK) which revealed its dependence on thiol reactivity and suggested it to have a cysteine protease activity. TLGST was inhibited with iodoacetamide indicated that residues of cysteine, histidine and methionine have significant impacts on structure and effectiveness of TLGST. Inhibition of TLGST with the metal chelating agent EDTA suggested it as a metalloenzyme. Triphenyltin chloride inhibited GSTs of German cockroach, *Apis mellifera macedonica* bee and equine [37, 40, 46], hematin inhibited that of filarial worm [2] while quercetin inhibited that of cockroach [46].

Here, *p*CMB was found the most potent inhibitor of TLGST since 1 mM inhibited 97.2% of its activity with an I50 = 0.7 mM (Fig. 4a). A straight line was achieved on plotting the Hill plot of TLGST inhibition with *p*CMB with a slope of 0.88 which refers to one binding site for *p*CMB on TLGST (Fig. 4b). A competitive inhibition of TLGST with *p*CMB was achieved due to absence of change in *Vmax* value while *Km* value was increased (Fig. 4c). The *Ki* value for inhibition of TLGST with *p*CMB is 0.3 mM (Fig. 4d) which confirms the potency of *p*CMB as TLGST inhibitor.

## Conclusion

In conclusion, this is the first report on purification and characterization of GST from larvae of the camel tick *H. dromedarii*. This TLGST might have an essential role in avoiding oxidative stress produced either by ingestion of large blood meals or via the broad utilization of pesticides. Targeting TLGST could be significant tool for development of prospective vaccines against ticks as a bio-control strategy to overcome the rapid grows in pesticide-resistant tick populations.

## Data Availability

All data and materials are available.

## Abbreviations

GST: Glutathione S transferase
*TLGST*: Tick larvae glutathione S transferase
*p*CMB: *p*-Chloromercuribenzoic acid
BSA: Bovine serum albumin
PAGE: Polyacrylamide gel electrophoresis
ROS: Reactive oxygen species
CDNB: 1-Chloro-2,4-dinitrobenzene
